# Biocompatibility Evaluation of an Artificial Metallic Bone with Lattice Structure for Reconstruction of Bone Defect

**DOI:** 10.3390/ma17174286

**Published:** 2024-08-29

**Authors:** Erika Yasuge, Tadashi Kawai, Shinsuke Kawamata, Isao Hoshi, Tadaharu Minamino, Shingo Kurosu, Hiroyuki Yamada

**Affiliations:** 1 Division of Oral and Maxillofacial Surgery, School of Dentistry, Iwate Medical University, 19-1, Uchimaru, Morioka 020-8505, Japan; erikay@iwate-med.ac.jp (E.Y.); kawashi@iwate-med.ac.jp (S.K.); isaohosh@iwate-med.ac.jp (I.H.); yamadah@iwate-med.ac.jp (H.Y.); 2 Department of Elementary Material Process Technology, Iwate Industrial Research Institute, 2-4-25, Kitaiioka, Morioka 020-0857, Japan; tada.minamino@gmail.com (T.M.); kurosu@pref.iwate.jp (S.K.)

**Keywords:** biocompatibility, mandibular reconstruction, mechanical stress, metallic artificial bone, lattice structure

## Abstract

Mandibular reconstruction for large bone defects is performed with consideration of patients’ specific morphology and sufficient strength. Metal additive manufacturing techniques have been used to develop biomaterials for mandibular reconstruction. Titanium artificial mandibles with a lattice structure have been proposed, and the optimal conditions for their strength to withstand mechanical stress around the mandible have been reported. This study investigated the biocompatibility of a titanium artificial bone with a lattice structure fabricated under optimal conditions. The samples were fabricated using metal additive manufacturing. Body diagonals with nodes (BDN) were selected as suitable lattice structures. Dode medium (DM) was selected for comparison. The samples were implanted into rabbit tibial defects and resected with the surrounding bone at two and four weeks. Specimens were evaluated radiographically, histologically, and histomorphometrically. Radiopacity in each lattice structure was observed at two and four weeks. Histological evaluation showed trabecular bone-like tissue inside the BDN compared to the DM at four weeks. No significant differences were noted in the bone volume inside the structures. This study demonstrated the in vivo compatibility of artificial metallic bones with a BDN structure under mechanical stress conditions.

## 1. Introduction

In the field of oral and maxillofacial surgery, large bone defects occur because of surgery for tumors, cysts, inflammation, or injury [1,2,3]. Reconstruction of these defects is necessary for functional and aesthetic restoration [4,5]. Titanium plates have been used for mandibular reconstruction [6]. However, there is a possibility of breakage due to insufficient strength, and restoration of aesthetics is difficult [7]. Free vascularized bone grafts, such as the fibula or scapula, are frequently used in large mandibular bone defects to achieve functional and aesthetic restoration [8]. However, the procedure is highly invasive, and functional disorders may possibly persist [9]. Recently, iliac particulate cancellous bone and marrow with custom-made titanium mesh trays have been adapted for mandibular reconstruction [10,11]. This procedure provides sufficient functional and aesthetic restoration and is less invasive than a free vascularized bone graft [12]. However, these materials have issues, such as titanium mesh tray fatigue and breakage [13]. In addition, these mandibular reconstruction methods have mechanical problems owing to the unique curved shape of the mandible and bite force. To resolve these problems, titanium mesh trays have been fabricated using metal additive manufacturing methods with computer-aided design data using selective laser melting or electron beam melting [14,15]. However, to the best of our knowledge, no established method provides sufficient strength and patient-specific reconstruction of the mandibular morphology.

To this end, research on materials using lattice structures is presently being conducted in the aerospace and orthopedic fields [16,17]. The lattice structure is a periodic cellular structure that is expected to be highly rigid and lightweight [18]. The type and periodic size of the unit cells in lattice structures affect the porosity and mechanical strength, and various unit cell structures, such as tetrahedrons, cubes, and gyroids, have been used in bone regeneration experiments as porous artificial materials [19,20]. If applied clinically, artificial mandibles with lattice structures are believed to proffer sufficient strength to withstand the biting force and to minimize the amount of grafted bone for bone defects. Recently, many lattice structures have been analyzed for strength against mechanical stress around the mandible using finite element analysis, and suitable lattice structures have been suggested for mandibular reconstruction as an optimal condition [21]. Notably, specimens fabricated by metal additive manufacturing using an electron beam melting system under the conditions from the finite element analysis are reportedly able to withstand biting force [21]. To apply this lattice-structured material to clinical mandibular reconstruction, it is necessary to confirm its affinity in vivo and its reaction with the surrounding tissues. It is expected that the lattice structure that can withstand mechanical stress around the mandible in mechanical tests will have a difference in the progression of new bone from the surrounding bone due to its strength, compared to a structure that cannot withstand mechanical stress. This study sought to investigate the biocompatibility of titanium artificial bone with a lattice structure fabricated by metal additive manufacturing using electron beam melting to develop a new method for mandibular reconstruction using an artificial metallic mandible that could withstand the bite force and minimize the amount of autologous bone grafting.

## 2. Materials and Methods

### 2.1. Selection of Lattice Structures

**Figure 1:**
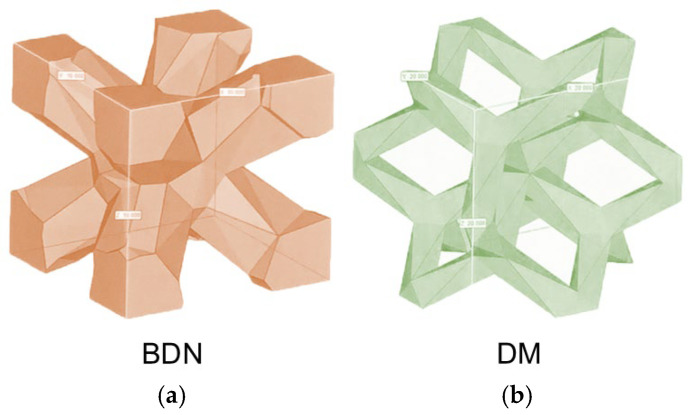
Images of lattice structures selected from the previous numerical material test. (**a**) BDN. A body-centered cubic lattice with nodes located at each vertex and the center of the cube. (**b**) DM. Lattice structures based on dodecahedra.

Based on a previous study, body diagonals with nodes (BDN) were selected for the lattice structure. The BDN structure satisfied the set Young’s modulus condition (10–30 GPa), the maximum bite force of 2000 N, and the fatigue strength that can withstand 6.7 million chewing cycles over 10 years, which indicated it could withstand mechanical stress around the mandible [21]. In addition, although the strength did not meet the requirements for withstanding mechanical stress around the mandible in the finite element analysis, the lattice structure of Dode medium (DM), which has a completely different structure, was selected for comparison. Figure 1 shows the images of the BDN and DM lattice structures. BDN is a body-centered cubic lattice with nodes located at each vertex and the center of the cube; because there is a fulcrum at the center of the diagonal, the structure constructed from its cells can be confirmed to have a constant isotropic Young’s modulus [22]. G10 is a two-dimensional honeycomb-like lattice in which three graphene structures are bonded to one cell, forming a hexagonal network [23]. Table 1 shows the results of the numerical material tests of the BDN and DM lattice structures [21].

### 2.2. Preparation of Implantation Samples

**Figure 2:**
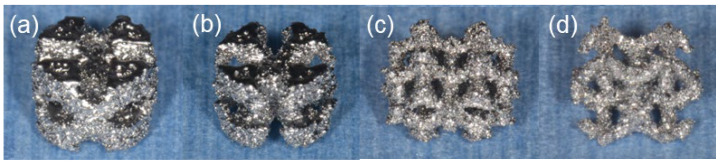
Images of each lattice structure sample with a diameter of 6 mm and a height of 4 mm. (**a**) BDN, cell size 3.5 mm (BDN3.5). (**b**) BDN, cell size 4.5 mm (BDN4.5). (**c**) DM, cell size 3.5 mm (DM3.5). (**d**) DM, cell size 4.5 mm (DM4.5).

The implantation samples were designed using computer-assisted design software (Materialize Magics 27; Materialize, Leuven, Belgium) [21] and prepared using an electron beam manufacturing system (Arcam EBM AX2; General Electric Company, Boston, MA, USA) [15]. Ti-64 grade 23 (AP&C, Boisbriand, QC, Canada) titanium alloy powder with a particle size of 45–100 μm (average particle size: 70 μm) was used for this study. The standard instructions recommended by the equipment manufacturer were used to determine the molding conditions of the samples. The surface properties of the metal samples prepared in this study have been reported previously [15]. The samples were cylindrical with a diameter of 6 mm and a height of 4 mm (Figure 2). In a previous study, the optimal unit cell size for the lattice structure was determined to be 3 mm [21]. However, in this study, the unit cell sizes were selected to be 3.5 and 4.5 mm, which are suitable for the fabrication of cylindrical samples (BND3.5, BND4.5, DM3.5, and DM4.5).

### 2.3. Mechanical Properties

In the previous study, the mechanical properties of BDN and DM samples were confirmed [21]. In compression tests on cylindrical samples of BDN and DM with a cell size of 3 mm, a diameter of 6 mm, and a height of 10 mm, the average 0.2% offset stresses were 194.8 ± 6.4 and 48.7 ± 1.3 MPa, respectively. In addition, in static bending tests on cylindrical samples of BDN and DM with a cell size of 3 mm, a diameter of 6 mm, and a height of 50 mm, the average 0.2% offset stresses were 498.0 ± 33.7 and 119.5 ± 13.3 N, respectively. In a fatigue test using a BDN with a cell size of 3 mm and a reference load of 175.6 N at the center of a cylinder with a diameter of 6 mm and a length of 40 mm, it was shown that the material would not break even after more than 10^6^ cycles at 12 MPa, which was approximately 4000 times the bite force of the molars in adults [24].

### 2.4. Animal

Male New Zealand white rabbits (body weight: 2.8–3.5 kg; Kitayama Labes Co., Ltd., Ina, Japan) were used for experiments. The study protocol followed the principles of laboratory animal care and national laws and was approved by the Animal Research Committee of Iwate Medical University (approval number: 04-029). Temperature and humidity were managed in the Iwate Medical University experimental facilities. The laboratory animals were bred in cages in the experimental facilities. The laboratory animals were tended by a contracted veterinarian, and the laboratory animals engaged in periodic activity and underwent regular examinations. After surgery, the animals were observed for the presence of any harmful phenomena such as wound compartment infection, decreased appetite, and death.

### 2.5. Implantation Procedures

**Figure 3:**
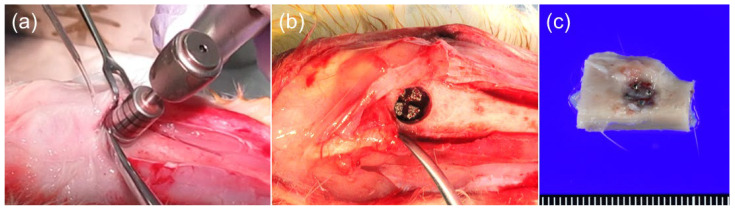
Images of the operation and resected specimen. (**a**) Bone defects were created in rabbit tibia using trephine bar. (**b**) Implantation of lattice-structure sample. (**c**) Lattice samples with surrounding bone were resected as specimen.

General anesthesia was administered initially with inhalation of 5% sevoflurane, followed by an intramuscular injection of a triple anesthetic mixture of 0.5 mg/kg medetomidine, 2.0 mg/kg midazolam, and 0.5 mg/kg butorphanol into the biceps femoris. Once the local anesthesia was administered by injecting 2% lidocaine with 1/80,000 epinephrine, the skin over both tibiae was incised, and the periosteum on both sides was ablated to expose tibiae from the mesial to distal ends. Bone defects (diameter: 6 mm, depth: 4 mm) were created at both the mesial and distal ends of the tibia using a trephine bar under continuous saline buffer irrigation. The lattice-structure samples were implanted into these trephine defects (Figure 3). As a negative control, no implantation was processed similarly. For each sample, the implantation site included both the mesial and distal ends. After the defects were treated, the ablated periosteum and skin were repositioned and sutured. The animals were euthanized two or four weeks after the operation with 100% carbon dioxide in a closed cage. The specimens were resected with the lattice structure sample and surrounding bone and fixed over one week using 10% formalin in neutral buffer solution (pH 7.4) at 4 °C. Three specimens from each group were prepared and analyzed.

### 2.6. Radiographical Analysis

Specimens were radiographed using a microradiography unit (Softex M-60S; Softex Co., Ltd., Ebina, Kanagawa, Japan) under standardized conditions (30 kV and 1 mA). Images were captured above the titanium sample. Subsequently, the specimens were scanned using micro-computed tomography (μCT) under standardized conditions (6 kV and 77 μA). After scanning, the image data were transferred to a three-dimensional image processing software program (myVGL ver. 2.2; VOLUME GRAPHICS, Heidelberg, Germany), and cross-sectional images of the specimen along the longitudinal axis obtained by image reconstruction were evaluated.

### 2.7. Histological and Quantitative Micrograph Analyses

The specimens were fixed in 70% ethanol, dehydrated in a graded series of ethanol, and embedded in methyl methacrylate (Technovit 9100; Nissin-EM Co., Ltd., Tokyo, Japan). Subsequently, they were sectioned using a low-speed saw (BS-300CP; EXAKT, Norderstedt, Holstein, Germany) with a diamond wafering blade. Sections were mounted onto plastic slides, ground, polished to 30–40 μm thickness, and stained with hematoxylin and eosin. Prepared sections were observed using a photomicroscope (ECLPSE 50i; Nikon Corporation, Tokyo, Japan), and images were captured using a digital camera (DS-Fil; Nikon Corporation). The percentage of bone area (bone%) was measured as the area occupied by bone tissue per unit area of space inside the lattice-structure sample. Bone% was measured from the digital images of the sections using ImageJ software (https://imagej.net/ij/index.html, 8 August 2024, National Institutes of Health, Bethesda, MD, USA).

### 2.8. Statistical Analysis

Statistical analysis was performed using BellCurve^®^ for Excel (Social Survey Research Information Co., Ltd., Tokyo, Japan). Histomorphometric data were analyzed using Excel version 10 software (Microsoft, Redmond, WA, USA). All values are reported as the mean ± standard deviation (SD). Mean differences between groups were compared using one-way analysis of variance, and significant differences were further analyzed using the Tukey multiple comparison post hoc test.

## 3. Results

### 3.1. Radiographical Analysis

**Figure 4:**
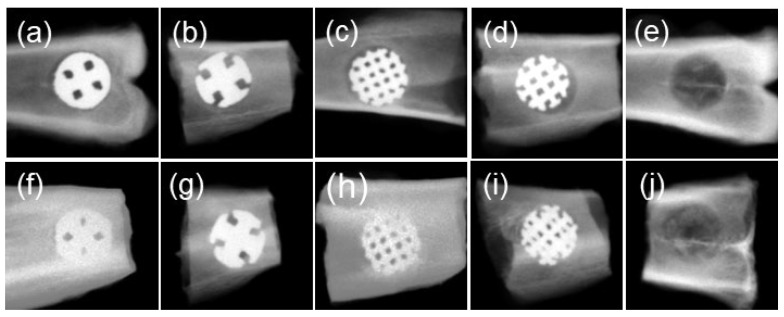
X-ray pictures of each specimen. View from directly above a cylindrical lattice structure sample. (**a**) BDN3.5, week 2. (**b**) BDN4.5, week 2. (**c**) DM3.5, week 2. (**d**) DM4.5, week 2. (**e**) Control, week 2. (**f**) BDN3.5, week 4. (**g**) BDN4.5, week 4. (**h**) DM3.5, week 4. (**i**) DM4.5, week 4. (**j**) Control, week 4. The radiopacity inside each lattice-structure sample increased by week 4. X-ray conditions were 30 kV and 1 mA.

The X-ray images are shown in Figure 4. This view showed the cylindrical lattice-structure sample directly from above. A slight radiopacity inside the lattice structure was observed in all lattice structure samples at two weeks. Bone defects were also detected in the control group. By four weeks, radiopacity inside the lattice structure had increased in all samples. Improved radiopacity was also observed in the control group. No resorption of the surrounding bone or fracture of the implanted lattice structure samples was observed after two or four weeks in any group.

**Figure 5:**
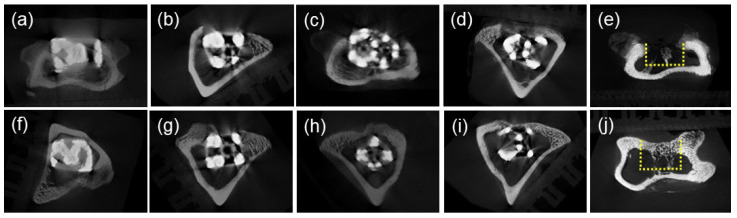
μCT images for each specimen. Horizontal view of the tibia. (**a**) BDN3.5, week 2. (**b**) BDN4.5, week 2. (**c**) DM3.5, week 2. (**d**) DM4.5, week 2. (**e**) Control, week 2. (**f**) BDN3.5, week 4. (**g**) BDN4.5, week 4. (**h**) DM3.5, week 4. (**i**) DM4.5, week 4. (**j**) Control, week 4. No absorption or fracture of the surrounding bone was observed at Weeks 2 or 4. Yellow dotted lines in control image indicate the original bone defect area.

The μCT images of the horizontal view of the tibia are shown in Figure 5. Evaluation of the interior of the lattice-structure samples was difficult due to the halation. After two and four weeks, no resorption or fracture was observed in the surrounding bone of any of the lattice-structure samples, and the embedded samples were in close contact with the surrounding bone. In the control group, slight radiopacity was observed in the cortical bone area at two weeks, which had increased at four weeks.

Radiographical analysis revealed no significant differences among the lattice-structure groups.

### 3.2. Histological Analysis

**Figure 6:**
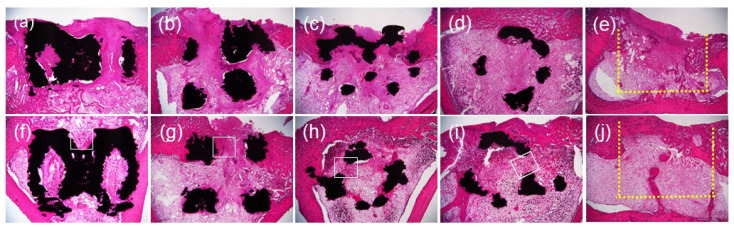
Images of the section of each hematoxylin and eosin-stained specimen. (**a**) BDN3.5, week 2. (**b**) BDN4.5, week 2. (**c**) DM3.5, week 2. (**d**) DM4.5, week 2. (**e**) Control, week 2. (**f**) BDN3.5, week 4. (**g**) BDN4.5, week 4. (**h**) DM3.5, week 4. (**i**) DM4.5, week 4. (**j**) Control, week 4. The space inside each lattice structure is filled with new bone and connective tissue by weeks 2 and 4. White squares indicate the area in Figure 7. Yellow dotted lines in control image indicate the original bone defect area. Magnification: ×2.

**Figure 7:**
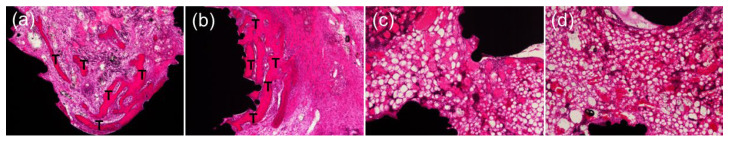
Magnified histological sections of each specimen at week 4. The areas shown in white squares in Figure 6f–i are indicated. (**a**) BDN3.5. (**b**) BDN4.5. (**c**) DM3.5. (**d**) DM4.5. In all cell sizes of BDN, more trabecular bone-like tissue was observed compared to that in the DM groups. T: trabecular bone-like tissue. Magnification: ×20.

Images of histological sections are shown in Figure 6. After two weeks, thin, new bone tissue was observed on the titanium surface within the lattice structure, and the spaces between the thin bones were filled with connective tissue in all lattice-structure groups. In the control group, the cortical bone defect remained, but connective tissue was formed and trabecular bone was partially formed. At four weeks, expansion of the bone tissue area was observed in all groups. In BDN of all cell sizes, more trabecular bone-like tissue was observed compared to that in the DM group (Figure 7). Cortical bone formation and internal healing were also observed in the control group. In all lattice structures, the internal spaces were filled with bone and connective tissues; few cavities were observed; and no inflammatory cell infiltration, fracture of the lattice-structure samples, or resorption of the surrounding bone was observed after two and four weeks.

### 3.3. Quantitative Micrograph Analysis

**Figure 8:**
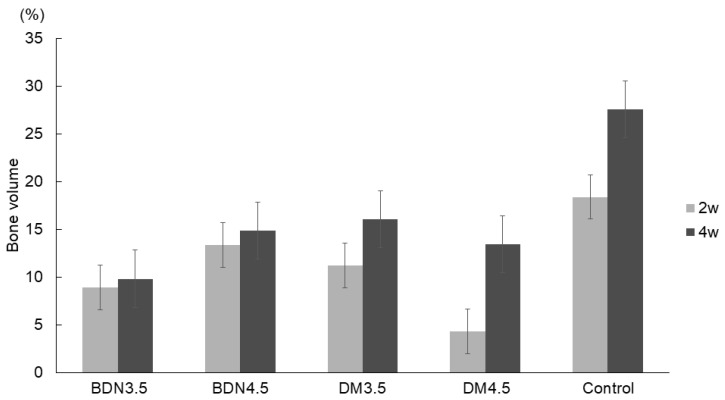
Quantitative micrograph analysis of the sections stained with hematoxylin and eosin. No significant differences were found between lattice-structure groups at weeks 2 and 4.

The results of the quantitative micrograph analysis are shown in Figure 8. The values of bone% ± SD in the BDN3.5, BDN4.5, DM3.5, DM4.5, and control groups at two weeks were 8.93 ± 3.10%, 13.48 ± 9.44%, 11.25 ± 5.27%, 4.35 ± 1.40%, and 18.42 ± 9.23%, respectively. After four weeks, these values were 9.8 ± 5.57%, 14.90 ± 9.30%, 16.09 ± 11.29%, 13.49 ± 6.64%, and 27.55 ± 4.83%, respectively. No significant differences were noted between the groups at two and four weeks. A slight increase in bone% was observed in each group from week 2 to week 4; however, this difference was not significant.

## 4. Discussion

In recent years, biomaterials fabricated using metal additive manufacturing have been developed in various fields to repair bone defects. Metal additive manufacturing can create patient-specific shapes and is effective in enhancing function [25]. In addition, the internal porosity allows for strong integration and long-term stability [25]. Comparison of an implant with a lattice structure to one without it reveals that the lattice-structure implant allows for a more uniform pressure distribution at the interface between the implant and bone [19]. In dental implants, the initial fixation can be strengthened by providing a lattice structure to the apical part [26]. As porosity is important, a method has been proposed to ensure that no errors during the additive manufacturing process prevent the creation of porosity [27]. In clinical practice, porous metal artificial bones have been implanted into the pelvis without causing complications such as infection or breakage [17]. In the field of oral surgery, the application of metallic artificial bones with a lattice structure mimicking the morphology of alveolar bone defects has been reported; however, their mechanical strength is yet to be evaluated [28]. The artificial bone with the lattice structure used in this study has been confirmed to have the strength to anticipate the mechanical stress around the mandible. This study demonstrates that a metallic artificial bone with a lattice structure that provides optimal conditions for mandibular reconstruction has an affinity for the in vivo environment.

Finite element analysis indicates that the BDM had a lattice structure that was robust against the mechanical stress around the mandible. Although the DM did not meet the strength criteria, it had a completely different structure from that of the BDN and was selected for comparison to avoid structural bias. In the present study, bone defects were created in rabbit tibiae. The rabbit mandible is thin; hence, it is difficult to create a lattice structure with a cell size of 3.5 mm using additive manufacturing to fit such a thin defect. Like the mandible, the tibia is subjected to mechanical stress, and therefore, it was believed to be suitable for evaluation in a dynamic in vivo environment.

In the X-ray findings, slight radiopacity was observed inside each lattice structure specimen at two weeks. After four weeks, an increase in the radiopacity of each structure was observed. The formation of hard tissue within the lattice structure suggests that bone growth from the surrounding bone was not prevented. Breakage of lattice structures was not observed; therefore, the strength of each lattice structure was sufficient. Although it was difficult to evaluate the interior of the lattice-structure samples in μCT scans because of halation, it was possible to evaluate the surrounding bone. No bone resorption or fracture was observed in the surrounding bone in any of the lattice-structure samples. It is believed that hard tissue formed within the lattice structure at an early stage, resulting in the initial fixation of the lattice-structure samples; therefore, no effect on the surrounding bone was observed.

Histological evaluation confirmed the formation of new bone and fibrous connective tissue within each lattice structure after two weeks. After four weeks, expansion of the new bone was observed. Furthermore, as no infiltration of inflammatory cells was observed, all lattice-structure samples were confirmed to be biocompatible. After four weeks, only BDN groups exhibited trabecular bone-like structures. Trabecular bone, an extension of the cortical bone into cancellous bone, shows an irregular 3D network formation in the marrow cavity and plays a role in bone-marrow formation [29]. Therefore, based on our previous study [21], because the lattice structure of BDN is stronger than that of DM, BDN may be more advantageous for bone formation in an environment subjected to mechanical stress. A quantitative micrograph evaluation revealed no significant differences between the lattice structures. Because new bone formation is due to the affinity between titanium and bone, it was believed that the rate of new bone formation inside the structure would not differ even if the cell structure was different. However, since trabecular bone-like structures were observed early in BDN, it is suggested that the conditions within the lattice structure are favorable for bone formation. Therefore, significant differences may be observed in long-term evaluations.

These results suggested that the lattice structure of BDN, which has been histologically confirmed to have a trabecular bone-like structure, was effective under mechanical stress. As expected in this study, it was demonstrated that artificial titanium materials with lattice structures that have different mechanical strengths also have different biocompatibility. It is thought that the mechanically strong lattice structure promotes the formation of bone tissue and other tissues by reducing mechanical stress on the inside of the structure. No significant histological or histomorphometric differences between cell sizes of 3.5 and 4.5 mm were noted; however, our previous report has suggested that smaller cell sizes are stronger. Therefore, based on the previous report [21] and the results of the present study, a BDN lattice structure with a cell size of 3.5 mm is suggested to be optimal for the fabrication of an artificial metallic bone with a lattice structure using additive manufacturing for mandibular reconstruction. However, the evaluation in this study was short-term and did not demonstrate long-term durability. Hence, it is necessary to confirm the long-term compatibility of the artificial metallic bone with the BDN lattice structure and to confirm whether the artificial metallic bone has become fatigued or fractured under a mechanical stress environment in vivo.

## 5. Conclusions

In this study, the biocompatibility of artificial metallic bone with a BDN lattice structure was confirmed in rabbit tibial bone defects. After four weeks, compared with the DM lattice structure, the new bone formed inside the BDN lattice structure showed a trabecular-like structure. The mechanical strength of BDN is considered superior to that of DM; therefore, it did not inhibit bone formation inside the lattice structure under the mechanically stressful environment of the rabbit tibiae. However, because this was a short-term evaluation, a long-term evaluation is necessary in the future.

## Figures and Tables

**Table 1:** Numerical material test of the BDN and DM lattice structures.

Structures	Equivalent Physical Properties (MPa)				Relative Density
	X Axis Direction	Y Axis Direction	Z Axis Direction	Average	
Body diagonals with nodes (BDN)	16,864	16,694	16,622	16,727	50.20%
Dode medium (DM)	630	630	630	630	12.60%

## Data Availability

The raw data required to reproduce these results cannot be shared at this time, as the data also form part of an ongoing study.
